# Nematicidal Amendments and Soil Remediation

**DOI:** 10.3390/plants9040429

**Published:** 2020-04-01

**Authors:** Nikoletta Ntalli, Zbigniew Adamski, Maria Doula, Nikolaos Monokrousos

**Affiliations:** 1 Department of Pesticides Control and Phytopharmacy, Benaki Phytopathological Institute, 8 S. Delta Str., 14561 Athens, Greece; 2 Electron and Confocal Microscope Laboratory, Faculty of Biology, Adam Mickiewicz University in Poznań, 61-614 Poznań, Poland; zbigniew.adamski@amu.edu.pl; 3 Department of Animal Physiology and Development, Faculty of Biology, Adam Mickiewicz University in Poznań, ul. Uniwersytetu Poznańskiego 6, 61-614 Poznań, Poland; 4 Laboratory of Non-Parasitic Diseases, Benaki Phytopathological Institute, 8 S. Delta Str., 14561 Athens, Greece; m.doula@bpi.gr; 5 Laboratory of Molecular Ecology, International Hellenic University, 57001 Thessaloniki, Greece; nmonokrousos@ihu.gr

**Keywords:** biofumigation, botanical amendment, *Meloidogyne*, nematode, organic fertilization, soil incorporation

## Abstract

The intensification of agriculture has created concerns about soil degradation and toxicity of agricultural chemicals to non-target organisms. As a result, there is great urgency for discovering new ecofriendly tools for pest management and plant nutrition. Botanical matrices and their extracts and purified secondary metabolites have received much research interest, but time-consuming registration issues have slowed their adoption. In contrast, cultural practices such as use of plant matrices as soil amendments could be immediately used as plant protectants or organic fertilizers. Herein, we focus on some types of soil amendments of botanical origin and their utilization for nematicidal activity and enhancement of plant nutrition. The mode of action is discussed in terms of parasite control as well as plant growth stimulation.

## 1. Introduction

Most currently employed agricultural systems employ the cultivation of large areas of land, thereby replacing the diversity of indigenous plants with cultivar-specific monoculture. These large-scale crop monocultures facilitate the increased prevalence and proliferation of diseases and pest insects [1], decrease the quantity and quality of soil organic matter, and deteriorate soil chemical and physical properties, thereby reducing soil fertility and crop productivity [2,3]. Soil amendment with botanicals may play a dual role in pest control and soil improvement, and can be more sustainable from an environmental perspective than the extensive use of synthetic pesticides and fertilizers. In this review, we describe different approaches or strategies to introduce botanical matrices into the soil either for management of pests and diseases or for soil remediation. In some cases, we discuss the mode of action for pest suppression by organic amendments in soil together with factors that contribute to efficacy, but a detailed description of the mechanism of action of any chemical substances involved is not presented. This review is structured into paragraphs addressing the use of botanical amendments as alternative plant protection and fertilization tools, followed by the amendment types employed. The main groups of nematode-suppressive amendments with related effects and references are reported in Table 1.

## 2. Botanical Amendments as Substitutes for Synthetic Nematicides

The most notorious below-ground agricultural targets are phytoparasitic nematodes; after their infection of plants, other soil borne pathogens often follow, such as *Fusarium* spp., *Phytophthora* spp., and *Pseudomonas* spp. The recently rated top 10 nematode pest genera include the root-knot (*Meloidogyne*), cyst (*Heterodera* and *Globodera*), and root lesion (*Pratylenchus*) nematodes [51]. Nematodes attack numerous crops and are responsible for estimated losses of more than EUR 157 billion per year [52]. On the other hand, free-living and non-phytoparasitic nematodes are ubiquitous, possess several beneficial roles in soils, and are considered good bioindicators for environmental monitoring because their populations are sensitive to environmental contaminants and because they are well classified into diverse functional groups [53,54]. Likewise, soil bacteria and fungi are important components of the functional biodiversity required to maintain sustainable agroecosystems [55]. Because preserving this essential soil biota is a specific protection goal in pesticide environmental risk assessment, recent studies have focused on the response of the soil microbial community to the so-called low-risk pesticide classes and botanically derived nematicidal preparations [56,57,58].

Indeed, great efforts are being made worldwide towards the development of more ecofriendly crop protection tools that protect non target organisms and other aspects of the environment. European Union legislation requires extensive experimental data for plant protection products prior to authorization, so as to avoid ecotoxicity concerns. For the most part, formulations of synthetic chemicals have been drastically restricted in usage because of their adverse environmental side effects and subsequent non-inclusion in Annex I of 91/414/EEC [59]. The most representative multipurpose soil sterilant, methyl bromide, has been unavailable since 2005 because of the Montreal Protocol, and no other compound has replicated its role in crop protection [60]. Moreover, new synthetic nematicides are expensive to develop, and inconsistent efficacy often renders the use of the currently available synthetic nematicides inadequate as a stand-alone pest control method. As a result, non-chemical methods to control soil pathogens and parasites are highly desirable, although such strategies also have limitations. For instance, solarization is expensive and may affect beneficial soil organisms, flooding cannot be performed in all locations and its efficacy depends on the crop and nematode species, and cultivar resistance to plant-parasitic nematodes may break under elevated temperatures and is species-dependent [61].

Most noteworthy is that many synthetic nematicides belong to the same chemical groups (e.g., organophosphates and carbamates) as many insecticides and acaricides, and they are often very toxic to soil microarthopods such as mites [62]. Many mites such as *Cosmolaelaps simplex*, a soil mite present in citrus orchards, prey on nematodes [63]. This mite hunts for plant-parasitic nematodes such as *Meloidogyne incognita* and the citrus nematode *Tylenchulus semipenetrans* and can significantly decrease their numbers [64]. Other predatory mites inhabiting soil and feeding on plant-parasitic nematodes include *Lasioseius penicilliger* [65], *Lasioseius subterraneous*, *Protogamasellus mica* [66,67], *Lasioseius scapulatus*, and *Gaeolaelaps aculeifer* [68]. Interestingly, the presence of organic manure generally increases the number of predatory mites in soil [69]. Consequently, the limited usage of synthetic pesticides and the application of other, ecofriendly methods may decrease plant infestation by nematodes, limit environmental pollution, and decrease the costs of plant production.

With respect to developing ecofriendly plant protection products, interest in botanical insecticides has surged since 2000 [70]. Countless types of plant secondary metabolites—including alcohols, aldehydes, fatty acid derivatives, terpenoids and phenolics—contribute independently or jointly to many biological processes. These metabolites may attract or repel nematodes, stimulate or inhibit egg-hatching, or exhibit nematicidal properties [71,72,73]. Nematicidal compounds naturally present in plants as products of secondary metabolism have been well documented in recent years [72,73,74,75]. Most importantly, these secondary metabolites form complexes that often act at multiple or novel target sites, thus reducing the likelihood of pest development of resistance [76,77]. Different natural molecules may affect directly nematode biology and behavior [71,78] but also interfere with respect to nematodes and other soil microfauna interaction, although this relationship needs extensive research. In this context, it has been found that volatile substances such as short-chain alcohols or aldehydes, acetate, or other secondary metabolites such as terpenes attract predators that feed on herbivores [79,80]. Last, toxicity to pests and pathogens may be provided by botanical amendments by virtue of their decomposition products, induced changes in soil physical and chemical properties, and their effects on biological antagonists [81,82,83,84].

## 3. Botanical Amendments as Substitutes for Synthetic Fertilizers

The use of fertilizers aims to equilibrate soil macronutrients diminished by monoculture. Despite the positive effects of inorganic fertilizers on crop yields since 1950 [85], they tend to be disfavored because they may lead to soil acidification, deficiency of nutrients, decreases in crop yields, and deterioration of fertility [86]. Inorganic fertilizers supplement the soil with nitrogen, phosphorous, and potassium in a plant-ready immediate manner, whereas organic fertilizers derived from plant materials slowly release nutrients as soil organisms decompose the organic matter under favorable soil temperature and humidity conditions. This slow-release method reduces the risk of nutrient leaching below the level of plant roots. Indeed, nutrient recovery efficiency for inorganic fertilizers is very important because it minimizes nutrient losses to the environment via surface runoff and leaching to groundwater [87]. A decade of organic fertilizer application was found to increase soil pH and organic C, the most often reported characteristic and important indicator of soil quality and agricultural sustainability [2,88]. Biofertilizers are products containing living microorganisms or natural substances that enhance soil chemical and biological properties, thereby stimulating plant growth and restoring soil fertility [89]. Indeed, because the soil is a primary recipient of waste products and chemicals, it needs physical (spray, vapor extraction, stabilization, solidification), chemical (photo-oxidation, dissolution, detergent use), and biological methods of remediation. Phytoremediation, the use of plants and/or their associated microorganisms to remove or render harmful material harmless, may also play a role in this direction [90]. The incorporation of plant residues into soil can be considered a keystone sustainability factor in improving soil structure and function by changing soil chemical properties and microbial community structures [91].

## 4. Botanical Amendment Type 1: Biofumigation Using Cover Crop Rotation and Incorporation

### 4.1. Plant Protection Properties Based on Chemical Factors

Although biofumigation is considered a non-chemical practice, it is in part based on the nematicidal activity of the plant secondary metabolites released in the soil and on the generation of nematicidal degradation byproducts [74,92]. In recent years, there has been a revival of desirable agronomic techniques such as crop rotation, soil organic matter content conservation, and the use of natural products in plant management and defense [93]. Brassica species form the oldest paradigm of plants employed in biofumigation [94], a term describing the utilization of volatile toxic compounds produced during decomposition of soil-incorporated tissue to combat soilborne diseases, pests, and weeds [4,95,96,97]. Upon the disruption of Brassicaceae tissues, the glucosinolates within are hydrolyzed by endogenous myrosinase, yielding bioactive compounds such as isothiocyanates, ionic isothiocyanate, organic cyanides, and nitriles [5]. These natural biofumigants are toxic to a range of soilborne pests and pathogens, including nematodes and fungi [75,98,99,100,101]. Interestingly, the Brassicaceae-based cover-crop/rotation practice effectively manages the top three rated economically important nematode pests, namely, root-knot, cyst, and lesion nematode, as well as others [6]. Along with Asteraceae species, the Brassicaceae have been long utilized to combat nematodes both in planta or in field experiments [7,8,17]; plant species in other botanical families used in the same way include the velvet bean (*Mucuna pruriens*), the castor bean (*Ricinus communis*), *Canavalia ensiformis*, and *Crotalaria* spp., as well as herbs [92].

The plant species that are usually considered as cover and rotation crops for biofumigation purposes include *Brassica oleracea* (broccoli, cabbage, cauliflower, kale); *Eruca sativa* (salad rocket, arugula); *Raphanus sativus* (radish); *Brassica napus* (canola, rapeseed); and various mustards such as *Sinapis alba* (white mustard), *Brassica rapa* (turnip), and *Brassica juncea* (mustard greens), although the latter two are good hosts for *M. incognita* and *Meloidogyne javanica* [4,9,10,11,12]. In a recent study, rapeseed, cabbage, and garden cress were good amendments, whereas mustard greens and flixweed (*Descurainia sophia*) consistently provided poor nematode management [4]. Similarly, the biocidal compounds released from ground, powdered preparations of *Sinapis alba*, *Brassica nigra*, *Brassica napus*, *Brassica rapa*, *Brassica juncea*, and *Brassica carinata* were toxic to the pathogenic fungus *Fusarium oxysporum* in vitro and in greenhouse experiments [102].

Care must be taken in that before selecting a plant for biofumigation purposes one should check the host status, as many species are hosts of important *Meloidogyne* spp. and thus might increase nematode populations rather than reduce them [103,104]. In particular, as stated above, Brassicaceous crops, although often advocated for nematode control, host a wide range of plant-parasitic nematodes [6]. As alternatives to green manures in these cases, seed meals or dried pellets can be used to avoid nematode population increases in the roots or tubers of the cover crops [105,106]. Moreover, an improperly selected plant species could reduce or harm populations of nematode species that prey on plant-parasitic nematodes or insects [107]. In this context, *Brassica* seed meal, a waste product of oil extraction, has been studied with respect to the effects of seed meal particle size on pathogen suppression and nontarget antagonism against soil nitrifying bacteria communities and their activities [108,109]. Conclusively and in accordance with Viaene and Abawi [110], a good cover crop is one that not only exhibits a poor host status to the target nematode pest species but also reduces the population of the nematode pest after incorporation of the crop into the soil. Interestingly, the use of plants yielding nematicidal compounds upon crop rotation and incorporation is more effective in controlling phytoparasitic nematodes than planting non-host plants lacking nematicidal compounds [111].

### 4.2. Plant Protection Properties Based on Other Factors

Biofumigation efficacy may additionally be based on the introduction of antagonistic microorganisms, the increase of plant tolerance and resistance, and the changes in soil physiology that render the soil unsuitable for phytoparasitic nematodes [92,101,112,113]. The suppression of soilborne pathogens and pests may actually result from their enhanced competition with copiotrophic soil microorganisms, which tend to accumulate after the addition of fresh organic matter [114,115]. Μany factors may increase the efficacy of bioamendments, such as the soil not being heavy enough and thus diminishing absorption of active molecules to clay, the growth stage at incorporation having maximum contents of pesticidal molecules, adequate soil moisture for plant decomposition and gas diffusion, and tarping with plastic film [92,116]. When soil temperature is increased by means of soil solarization, nematode control efficacy of broccoli bioamedments increases [117]; additionally, the anaerobic conditions improve control of soil pathogens [118,119,120]. The decomposition of amendments in saturated soil produces short chain fatty acids and fermentation products of carbon-rich organic matter, including acetic, butyric, formic, and propionic acids, which are highly nematicidal [92,121,122]. Additionally, under such conditions, oxygen is depleted by bacteria, and redox potential and pH are decreased by the organic acids generated from amendments [123]. In turn, the low soil pH deactivates soil bacteria capable of oxidizing the organic acids, which in turn become more persistent and thus more active through time [124]. During soil mineralization, soil microbes modify the nitrogen found in organic matter from ammonia (NH_3_) to ammonium salts (NH_4_^+^). Soil nitrogen in the form of ammonia (NH_3_) negatively affects root-knot nematodes [125]. Exposure of *Meloidogyne javanica* larvae to 9.3 µg mL^−1^ ammonia for 40 h in vitro was lethal to 95% of the nematode population. Unlike NH_3_, which translocates across cell membranes easily, ammonium (NH_4_^+^) crosses cell membranes slowly at best, and ammonium in *Bacillus cereus* culture filtrates had no effect on nematodes [126]. The soil pH determines the equilibrium of ammonia and the non-nematicidal ammonium contents in the soil liquid. Although temporarily increasing pH in alkaline soils with low buffering capacity is one potential way of increasing ammonia release [92,127], ammonia is not stable in time. Microorganisms oxidize it to nitrite (NO_2_^−^) and then nitrate (NO_3_^−^) in a process called nitrification. Thus, combined applications of nitrification inhibitors with ammonia-releasing organic amendments may expand nematicidal activity [128]. Examples of such nitrification inhibitors include synthetics such as nitrapyrin or naturally based substances such as *Azadirachta indica* seed kernel powder, the furanoflavonoid karanjin from the seeds of *Pongamia pinnata*, or essential oils [129,130,131]. Combining organic amendments with soil tarping may enhance nematicidal activity because the ammonia is physically retained in soil for longer periods and diffuses into deeper soil levels due to the elevated temperature and vapor pressure [92].

### 4.3. Secondary Beneficial Properties on Soil Microbes and Saprophytic Nematodes

Amending the soil with organic matter directly improves soil by ameliorating the soil nutrient status and water-holding capacity and increasing the presence and action of beneficial soil organisms, including antagonists of plant-parasitic nematodes [132]. Although *Brassica* species are mainly studied and patented for their plant-protective features mainly attributed to the isothiocyanates [133], they may additionally provide secondary beneficial effects such as enhancing soil microorganism numbers, preserving beneficial organisms, extending the residual life of biological activity, increasing plant growth [74], reducing the ecological footprint of agriculture [134], and improving soil structure [135]. In this context, it is well known that bacteria, fungi, and free-living nematodes are the main drivers of nutrient cycling, decomposition of organic residues, formation of humic substances, and pollutant degradation [93,136,137], whereas free-living nematodes grazing on bacteria and fungi regulate decomposition and nitrogen mineralization in the soil ecosystem [93,138,139,140]. For instance, the application of cut tissues from various Brassicaceae species, for instance *B. oleracea*, *Descurainia sophia*, *Lepidium sativum*, *Sinapis alba*, and *B. napus*, increases soil organic matter and subsequently soil microbial biomass, thus resulting in elevated essential mineral and nitrogen concentrations, whereas organic fertilization with *Raphanus sativus* was found to lessen nitrogen leaching [4,141].

Limited research has examined the effects of cover crops on above- and belowground communities of organisms [142]. Free-living nematodes may be assigned to five trophic groups: bacterivores, fungivores, non-parasitic plant feeders, omnivores, and predators [143]. Nematodes in the soil food chain range from fast-growing, fast-breeding bacteria-feeding nematodes at the bottom (colonizers) to slow-growing, slow-reproducing predatory nematodes (persisters) at the top. Legumes, forbs, and grasses have dissimilar rooting patterns and thus create habitats more congenial to some species of nematodes than others [144]. On the other hand, cover crop biochemical compositions including the carbon to nitrogen (C/N) ratio affect the N release from cover crop residues and thus free-living nematode fauna [145]. Thus specific nematode genera increase or decrease on the basis of the plant species used as cover crop and/or green manure [146]. For instance, soil incorporation of *Crotalaria juncea* increases nematode-trapping fungi, fungi parasitic on *Rotylenchulus reniformis* eggs, vermiform stage parasites, and bacterivorous nematodes more proficiently than incorporation of *Tagetes erecta* or *Brassica napus* [13]. In another study [147], *C. juncea* soil amendments increased the numbers of omnivorous and predatory nematodes but the *M. incognita* population remained the same. *Crotalaria juncea* has characteristics that may make the crop useful for nematode antagonism; for example, *C. juncea* seems to be associated with soil rhizospheric enzymatic activity correlated with microbial activity and suppressiveness to soilborne plant pathogens [148]. Exudates from *Crotalaria* spp. select for microbial species antagonistic to phytopathogenic fungi and nematodes [149]. In general, the presence of omnivorous and predatory nematodes indicates that the soil food web is diverse and stable; moreover, their high abundance means that the soil has some capacity to suppress populations of phytoparasitic nematodes and other soilborne pathogens [138]. When *Synapsis alba* green manure was used as a biofumigant, it resulted in an increase in bacterivorous nematodes and a decrease in fungivores, but no change in populations of omnivores and predators occurred [14]. Decreases in fungivores after green-manure application of Brassicaceae crops is advocated for biological soil disinfestation or increasing nematode antagonists [14]. It has to be noted that predatory nematodes belonging to the orders Dorylaimida, Mononchida, and Diplogasterida suppress phytoparasitic nematodes [15].

Crop rotation and incorporation can also affect soil bacteria populations, which in turn may affect phytoparasites. There are many reports on the nematicidal activity of plant growth-promoting rhizobacteria (PGPR) colonizing different cover crop rhizospheres [150,151,152,153]. PGPR produce a wide range of plant growth substances, for instance gibberellins, auxins, cytokinins, and ethylene [154], and in combination with composts PGPR limit pest damage to plants [155]. Additionally, PGPR enhance the uptake of phosphorous and nitrogen by plants through nitrogen fixation and phosphate solubilization [156,157]. With respect to plant pathogens, 39 isolates of rhizobacteria recovered from different economic plants produced hydrogen cyanide and thus inhibited growth of *Agrobacterium tumefaciens* and affected viability of *Meloidogyne incognita* juveniles in vitro [158]. In addition, rhizobacteria from the roots of antagonistic plants, such as velvet bean, castor bean, and rye, were mainly Gram-negative genera and reduced significantly nematode damage on soybean by *Heterodera glycines* and *M. incognita* compared to rhizobacteria isolated from the soybean rhizosphere [159]. Sturz and Kimpinski [18] suggested that use of the endophytes *Microbacterium esteraromaticum* and *Kocuria varians* isolated from *Tagetes* sp. can play a significant role depressing the population of root-lesion nematodes (*Pratylenchus penetrans*) in potato fields. However, microbial communities respond differently to the bioactive secondary metabolites produced after soil incorporation of different botanicals, such as with respect to type and content of glucosinolates and isothiocyanates subsequently generated [160]. For instance, Alphaproteobacteria were found to be much more sensitive to glucosinolates than other groups [161], and ascomycetes were more sensitive to ammonia-oxidizing bacteria [162]. On the other hand, the addition of organic matter to soil may affect the microbial community and pathogen incidence without any direct effect from the isothiocyanates produced by seed meal soil amendment [163].

Finally, cover crops may host nematode-trapping and other fungi that attack nematodes; cover crop incorporation into soil may affect indirectly their abundance. Nematode-trapping fungi are a major group of nematode antagonists that can be enhanced by incorporation of residues of *C. juncea* [16]. In that direction, *Crotalaria juncea*, *Brassica napus*, and *Tagetes erecta*, intercropped with *Ananas comosus* to control *Rotylenchulus reniformis*, developed significant population densities of nematode-trapping fungi [13]. Linford et al. [164] found that incorporation of chopped pineapple plants increased populations of bacteria, bacterivorous nematodes, and nematode-trapping fungi, which in turn attacked root-knot nematode juveniles. The effects of organic amendments on nematode-trapping fungi and nematode suppression may also highly depend on fungal species and on the type and quantity of the amended organic matter [92]. The application of organic matter from *Tagetes minuta*, *Ricinus communis*, and *Datura stramonium* stimulated the parasitic activity of *Paecilomyces lilacinus* against *Meloidogyne javanica* eggs [19]. Lastly, Sturz and Kimpinski (2004) [18] reported that endoroot bacteria from marigold *Tagetes erecta* can play a role in of *Pratylenchus penetrans* suppression through the attenuation of nematode proliferation.

## 5. Botanical Amendment Type 2: Recycling Wastes for Use as Chars and Composts

### 5.1. Plant Protection Properties

Another practice that involves the introduction of botanical matrices into the soil employs agricultural wastes recycled into chars or composts. In fact, an ideal economic model is a circular one wherein resource consumption and waste formation are minimized through the management and reutilization of wastes. Consequently, the use of solid olive wastes to make biochars for agricultural utilization fits well in the initiatives of the European Waste Framework Directive (Directive 2008/98/EC, 19 November 2008) [20]. Pyrolysis is the procedure of anaerobic thermal decomposition performed at high temperatures leading to the manufacture of solid products (chars). “Biochars” are chars used as soil amendments to improve plant productivity, preserve carbon reserves, or filter percolating soil water [20], but they also exhibit pesticidal properties. For example, when different biochar extracts were tested, the root growth of *Lepidium sativum* and *Brassica rapa* and the survival of *Meloidogyne incognita* juveniles were inhibited by the thermally untreated material or biochar produced at 300 °C, but toxicity decreased at higher pyrolysis temperatures [20]. Similarly, growth of the fungi *Aspergillus*, *Fusarium*, *Rhizoctonia*, and *Trichoderma* was stimulated by the organic feedstock but were inhibited by the thermally treated biochars [20]. In addition, Huang et al. [21] showed that the incorporation of wood biochar reduced the susceptibility of *Oryza sativa* to *Meloidogyne graminicola*, and Ikwunagu et al. [22] reported that the application of bitter leaf biochar significantly inhibited *Meloidogyne* spp. egg hatch in a *Vigna radiata* experiment.

Composting is another tool for the management of municipal and agro-industrial wastes. It is the biological stabilization of solid plant biomass or animal residues and is performed at 45–65 °C to eliminate pathogenic microorganisms. The organic material decomposes aerobically to yield compost rich in available nutrients and able to improve the physical and chemical properties of the soil if incorporated, thereby ultimately increasing crop yield [23]. Earthworms such as *Eisenia fetida* can be used to accelerate the process, in which case the material passes through the gut and undergoes transformation [165]. Interestingly, the compost incorporated into soil exhibits activity against plant soil-borne pathogens and pests, including plant-parasitic nematodes [166]. According to De Corato et al. [24], it is important to understand the taxonomic structure of the compost’s microbial consortium to determine the species potentially involved in suppression. In this investigation, significant suppressive effects concurrently occurred against *Pythium ultimum* (damping-off of cucumber), *Rhizoctonia solani* (damping-off of bean), *Phytophthora nicotianae* (root rot of tomato), and *Verticillium dahliae* (wilt of eggplant) upon amendment of municipal-solid-waste composts with extracts obtained from green composts [24]. Interestingly, when *Trichoderma harzianum* was used as a decomposer for neem, cassava, sawdust, and *Tithonia* compost preparation, each compost suppressed populations of *Meloidogyne*, *Heterodera*, *Tylenchus*, and *Tylenchulus* species and significantly increased the growth and yield of okra plants [25]. Forge et al. [26] reported that compost used as a preplant soil amendment in red raspberry fields suppressed *Pratylenchus penetrans* populations for two growing seasons. These results were comparable to those recorded in fields under fumigation treatment.

When vermicompost from municipal wastes was used alone or mixed with urea, the development and survival of two potato-cyst nematodes (*Globodera rostochiensis* and *G. pallida*) significantly decreased according to the number of cysts, eggs, and juveniles; additional positive effects on stem weight and stem height occurred [27]. The fungus *Fusarium oxysporum*, which severely restricts global tomato yield, was sufficiently controlled by the use of vermicompost in long-term continuous tomato cropping. In particular, a strong positive relationship existed between the relative abundance of some bacterial groups (including the genera *Nocardioides*, *Ilumatobacter*, and *Gaiella*) and *Fusarium* wilt inhibition [45]. In a 3-year experiment comparing organic farming involving compost of *Tithonia diversifolia* and neem cake (*Azadirachta indica*); conventional farming involving fertilizer and nematicide; farmer practice involving manure, *Tithonia diversifolia*, and wood ash; and a farm with no input application, the plant-parasitic nematodes including *Pratylenchus* and *Meloidogyne* were significantly reduced in the organic system compared to the three others [62] and for a longer period. As well as this, Neher [46] concluded that conventional farming was less effective than organic farming in suppressing phytoparasitic nematodes. She correlated positively the indices of plant-parasitic nematodes with concentrations of nitrate. Although synthetic nematicides may provide an immediate toxic effect upon phytoparasitic nematodes, organic amendments act for longer periods [57] via chemicals released and their degradation products and by favoring nematodes, fungi, and bacteria preying upon or parasitizing the plant-parasitic nematodes [92,167,168]. Additionally, organic amendments increase plant tolerance to phytoparasitic nematodes, and even though in some investigations the nematode numbers were not affected by the incorporated organic material [112,169,170], yields significantly increased. Moreover, as described in previous paragraphs, organic farming practices may alter parameters related to soil structure and characteristics (e.g., pH, salinity, particle aggregation) and thus affect plant-parasitic nematodes indirectly [92]. In most of the cases the aforementioned mechanisms may act simultaneously [17].

There are some reports on the predation of mites on nematodes, including mites belonging to the orders Mesostigmata, Oribatida, and Gamasina. Rodriguez et al. [171] observed nymphs of Mesostigmatic mites, although not the adult stages of *Macrocheles muscaedomesticae*, preferably preying on *Rhabditella leptura* nematodes, rather than housefly eggs or nematodes of the genera *Diplogaster* and *Panagrolaimus*. Interestingly, the low preference for *Diplogaster* would be positive for phytoparasitic nematode control, as *Diplogaster* preys on juveniles of *Meloidogyne javanica* and *Tylenchulus semipenetrans* [28]. It has to be noted that *Macrocheles muscaedomesticae* is a phoretic mite, frequently found on dung and compost piles, where it feeds on nematodes and other organisms [29,30,31]. Organic manures and composts are regarded as microscale hot spots of biodiversity, where oribatid or mesostigmatic mites can frequently be found [32], such as the oribatid *Scheloribates* spp. [33]. Mites of this genus feed intensively on nematodes, including the *M. incognita* invasive J2 stage, which can be consumed at an estimated rate of 60 J2 per day [34]. This observation is doubly important, as agroecosystems may be less rich in microarthropods, including mites, compared with natural biocenoses [35,36,37], and composting increases their abundance [38]. Additionally, a harmful effect of compost on microarthropods was not observed [37]. Therefore, composting may not only affect nematodes directly, but also increase the abundance of predatory mites. Overall, macrochelid mites feed on members of the nematode family Rhabditidae and others, including the following genera: *Panagrellus*, *Aphelenchus*, *Aphelenchoides*, *Diplogasteroides*, *Diplogaster*, *Panagrolaimus*, *Rhabditis* (*Rhabditella*), and *Rhitis* [39,40,41]. As well as this, *Protogamasellus mica* (cohort Gamasina), found on sugarcane fields, appeared to be an economically important predator of *Meloidogyne javanica*, *Pratylenchus zeae*, and *Tylenchorhynchus annulatus* [66]. *Tyrophagus putrescentiae* (Acaridae) was reported as a dominant mite in organic manures [42]. Although the species is regarded as a pest of stored products, it was found as an intensive predator of invasive stages of some plant-parasitic nematodes: *M. incognita*, *Anguina tritici*, and *Heterodera mothi*, and a moderate predator upon many other species [43]. Greater investigation should be conducted on the role of predatory mites on phytoparasitic nematode suppression. All these data provide further support for the usage of compost and organic manure as soil amendments.

### 5.2. Soil Enhancement Properties

Biochar acts as a soil improver and conditioner positively affecting soil chemical, physical, and microbial properties. The use of biochar can improve soil structure and increase soil porosity, electrical conductivity, organic matter, and cation exchange capacity [44,172,173]. Additionally, biochar increases soil microbial community composition and biomass as well as soil enzyme activity [174,175,176,177]. The main advantages of biochar are its high nutrient content and its function as a slow-release fertilizer, thus affecting plant growth for longer periods than conventional fertilizers (organic or inorganic). Composts may be used as soil enhancers also. As with cover and rotation crops, composts are important sources of organic matter and provide the soil with required macro- and micronutrients [92]. Like biochars, composts are decomposed slowly in the soil; thus, nutrients remain for a longer time, thereby conserving the microbial population, which remains active for a longer time [178]. The use of vermicompost in a long-term continuous tomato cropping soil positively altered the soil pH, NH_4_^+^-N, soil organic matter, and dissolved organic carbon [45]. Organic amendments have been found to improve the nine indicators of soil health: aggregate stability, water-holding capacity, infiltration/porosity, erosion/runoff, nutrient cycling, organic carbon, microbial biomass, macrofauna abundance, and weed seed bank. Furthermore, combinations of organic amendments such as composts, manures, and vermicomposts improve soil health metrics, carbon, nitrogen (N), and aggregate stability [179], as well as the soil microbial biomass [180]. In another study [160], different Brassicaceous seed meal amendments stimulated nitric oxide (NO) generation in orchard soil, thus proving that Brassicaceae residues efficiently provide plant-available nitrogen. In contrast, the microbial communities and overall soil functions responded differentially to both seed meal type/glucosinolate content and isothiocyanate generation [160]. According to the literature, during the maturation phase of composting the fungal populations are predominant, whereas bacteria populations decrease due to the reduction of substrate quality. In mature composts, however, competition and/or synergic actions are activated [181]. Vida et al. [182] examined the beneficial effects of composted almond shells in an avocado orchard and observed that the abundance of γ-Proteobacteria increased after compost treatment. Members of γ-Proteobacteria are capable of fixing nitrogen in the soil and help in the oxidation of sulfur, iron, and methane [183].

## 6. Botanical Amendment Type 3: Other Products

### 6.1. Plant Protection Properties

Various botanical materials, not manufactured in an industrial level, been tested as plant protectants or soil improvers. For example, soil incorporation of raw garlic stalk in eggplant monoculture significantly increased soil electrical conductivity (EC), N, P, K, and enzyme activity. The soil pH significantly decreased, whereas beneficial bacteria belonging to Anaerolineaceae, Acidobacteria, and Cyanobacteria increased in abundance. Interestingly, the pathogenic fungal genera *Fusarium* and *Acremonium* decreased [91]. In another study, neem cake, mustard cake, and groundnut cake controlled *Fusarium oxysporum* in vitro [184]. When dried leaves of *Cannabis sativa* and *Zanthoxylum alatum* were incorporated into the soil to control *Meloidogyne incognita*, *C. sativa* was significantly better, and maximum improvement in growth parameters occurred, whereas nematode infection and reproduction diminished [47]. In some cases, phytotoxicity concerns must be considered, as in the case of orange peel meals, which provided good nematicidal activity against root-knot nematodes but were toxic to tomato plants [48]. Cuphea plant, marigold, pennycress seed powder, and canola meal, all aboveground plant parts except seeds, were effective against soybean cyst nematode, *Heterodera glycines*; populations were significantly reduced compared to controls. These amendments contain chemical compounds that are phytotoxic as well as nematotoxic [49]. Of course, reductions in final yield must be ascertained before the agronomic importance of the potential phytotoxicity becomes established [49].

### 6.2. Bio-Fertilizer Properties

In a pot experiment performed by our group, we evaluated the nematicidal effects of *Pimpinella anisum*, *Petroselinum crispum*, and *Eruca sativa* as self-prepared soil amendments for tomato plants. All three botanicals significantly controlled nematodes, whereas parsley and rocket also had a positive effect on tomato root growth. In addition, all botanical treatments enhanced soil microorganisms in terms of both biomass and functionality, as indicated by urease and β-glucosidase activities; moreover, populations of bacterivorous nematodes increased [58]. Likewise, a *Melia azedarach* fruit powder suppressed *Meloidogyne* spp. under greenhouse conditions often at the same levels as the commercial nematicide oxamyl and, more importantly, enhanced soil biological activity, as indicated by the proliferation of soil microbes and microbial feeding nematodes. On the contrary, oxamyl adversely affected the soil community, especially the free-living nematodes [57]. The highest soil deterioration was similarly reported for commercial nematicides (e.g., metam-sodium) by other researchers who compared them with defatted seed meals of Brassicaceous tissues. Interestingly, fumigation affected the microbial community composition to a greater extent than its total abundance, suggesting a pronounced resilience of soil biological communities after fumigation [93]. According to Trivedi et al. [50], each soil aggregate represents a different ecological niche for microbial colonization. The physico-chemical conditions in specific aggregate fractions differ from the established conditions in the bulk soil and clearly influence the microbial communities in soils [185]. As a result, the specific effects of cover crops on the bacterial and fungal communities may vary among different spatial habitats in the soil [186]. Most interestingly, when a “static fertilization experiment” was performed to study the response of soil microbial communities to 113 years of different fertilization regimes (mineral, organic, and combined fertilization), the application of farmyard manure in combination with mineral fertilizer resulted in high crop yield attributed not only to the increased soil nutrient contents but also to the enrichment of beneficial microorganisms [187]

## 7. Conclusions

In an effort to discover dual pest management and fertilization tools with limited negative or even positive environmental impact, we reviewed the botanicals. In the long term, it seems difficult to achieve management of pests by using a single strategy such as application of pesticides, host plant resistance, crop rotation, and/or other methods [188]. In addition, the withdrawal of many plant protection products from world markets renders mandatory the integration of alternative agricultural pest control methods [74]. Reducing population levels of economically important pests by using pesticidal follow-up crops in infested fields improves the overall structure of the soil together with its physical and chemical properties [189]. Obviously, cover and rotation crops with rich amounts of bioactivity and poor host status should be preferable to avoid pest buildup, and procedures increasing efficacy like soil tarping may be employed. Finally, the search for more inexpensive plant protection and fertilization candidate plants could worthwhile; these plants could be readily available even in resource-poor countries and would be valuable if they exhibit pesticidal or soil-improving properties.

## Figures and Tables

**Table 1:** Main groups of nematode-suppressive amendments with related effects and references.

Botanical Amendment Type	Categories	Plant Species/Type of Waste	Effect	References
Biofumigation, cover-crop rotation, and incorporation	Brassicaceae species	*Brassica oleracea, Brassica napus, Brassica rapa, Brassica juncea, Eruca sativa, Raphanus sativus, Sinapis alba*	Release of isothiocyanates, ionic isothiocyanate, organic cyanides, and nitriles increase the nematode-trapping fungi or parasitic fungi, or other nematode antagonists (e.g., mites)	[4,5,6,7,8,9,10,11,12,13,14,15,16]
	Asteraceae species	*Tagetes erecta, Tagetes minuta, Tagetes* sp.	Increase of nematode-trapping fungi or parasitic fungi, activity via endophytes and parasitism	[13,17,18,19,20]
Recycling wastes	Biochars	Biochar extracts, wood biochar, bitter leaves biochar	Inhibition of survival of root knot nematodes, reduced plant susceptibility, and egg hatchability	[21,22,23]
	Composts	Municipal-solid waste with extracts obtained from green composts	Increase of predatory mites and fungi or bacteria that control the nematodes	[24,25,26,27,28,29,30,31,32,33,34,35,36,37,38,39,40,41,42,43,44]
	Vermicomposts	Municipal wastes	Decreased number of cysts, eggs, and juveniles of cyst nematodes	[45,46]
Self-made products		Dried leaves of *Canabis sativa*	Chemical compounds reduce RKN infection and reproduction	[47,48,49]
		Orange peel meals	Chemical compounds decrease RKN numbers	
		Cuphea plant, marigold, pennycress seed powder, and canola meal	Release of nematotoxic compounds that reduce cyst nematode numbers	[50]
